# Longitudinal Follow-Up Study on the Side Effects of COVID-19 Vaccines: A Telephonic Questionnaire Approach

**DOI:** 10.7759/cureus.62917

**Published:** 2024-06-22

**Authors:** Krishna Priya Jakkula, J Kishore, Uma Maheswar Rao

**Affiliations:** 1 Medicine, Lions Cancer Hospital, Visakhapatnam, IND; 2 General Surgery, Lions Cancer Hospital, Visakhapatnam, IND

**Keywords:** logistic regression analysis, telephonic questionnaire, longitudinal study, demographic characteristics, side effects, covaxin, covishield, covid-19 vaccines

## Abstract

Background: The COVID-19 pandemic, caused by severe acute respiratory syndrome coronavirus 2 (SARS-CoV-2), has prompted urgent efforts to develop and deploy effective vaccines. Covishield and Covaxin are two prominent COVID-19 vaccines authorized for emergency use; however, concerns regarding their safety persist.

Objective: This longitudinal follow-up study aimed to comprehensively assess and compare the demographic characteristics, frequencies, severities of reported side effects, and associations between vaccine type and demographic factors among individuals vaccinated with Covishield and Covaxin.

Methods: A telephonic questionnaire was used to collect data from individuals who attended COVID-19 vaccination programs between January 1, 2021, and January 1, 2022. Logistic regression analysis was performed to investigate the associations between vaccine type, demographic factors, and likelihood of experiencing side effects.

Results: Covaxin recipients exhibited a lower incidence of mild flu-like illness (16 cases) and post-vaccination infection (55 cases) than Covishield recipients (110 and 98 cases, respectively). However, Covaxin recipients reported more cases of soreness at the injection site (139 cases) than did Covishield recipients (172 cases). Logistic regression analysis revealed significantly higher odds of experiencing side effects among Covaxin recipients than among Covishield recipients (OR = 1.687, p < 0.001). Age was inversely associated with the likelihood of experiencing side effects (OR = 0.982, p < 0.001), while sex and ethnicity also exhibited significant associations.

Conclusion: This study provides valuable insights into the safety profiles of the Covishield and Covaxin COVID-19 vaccines. These findings underscore the importance of ongoing surveillance and evaluation of vaccine safety and tolerability to inform public health policies and vaccination strategies.

## Introduction

The global COVID-19 pandemic caused by severe acute respiratory syndrome coronavirus 2 (SARS-CoV-2) has spurred an urgent need for effective vaccination strategies to curb transmission and reduce the disease burden [1,2]. Vaccination remains the cornerstone of public health efforts to control the spread of infectious diseases, and the development and deployment of COVID-19 vaccines have been pivotal in combating the pandemic [3]. Among the vaccines authorized for emergency use, Covishield (ChAdOx1 nCoV-19/AZD1222) and Covaxin (BBV152) have gained prominence, contributing significantly to vaccination campaigns worldwide [4]. Covishield, developed by AstraZeneca and the University of Oxford, is a viral vector vaccine based on a chimpanzee adenovirus (ChAdOx1) vector encoding the SARS-CoV-2 spike protein [5]. Covaxin, developed by Bharat Biotech in collaboration with the Indian Council of Medical Research (ICMR), is an inactivated whole-virus vaccine that induces an immune response against multiple viral proteins [6].

While both Covishield and Covaxin have demonstrated efficacy in clinical trials, concerns regarding their safety profiles persist, necessitating ongoing monitoring and evaluation [7]. Adverse events following immunization (AEFI), including local reactions and systemic symptoms, have been reported at varying frequencies following COVID-19 vaccination [8]. Understanding the comparative safety and tolerability of Covishield and Covaxin is crucial for optimizing vaccination strategies and ensuring public confidence in immunization programs. Given the widespread use of Covishield and Covaxin in COVID-19 vaccination campaigns, it is imperative to comprehensively assess and compare their safety profiles. Understanding the demographic characteristics, frequencies, and severities of the reported side effects associated with these vaccines is essential for informed decision-making by healthcare providers, policymakers, and vaccine recipients.

The objectives of this study were as follows: first, to assess and compare the demographic characteristics of individuals who have received Covishield and Covaxin vaccinations; second, to evaluate the frequencies and severities of reported side effects associated with both vaccines; third, to investigate the associations between vaccine type, demographic factors, and the likelihood of experiencing side effects; and finally, to provide evidence-based recommendations for vaccine selection and immunization strategies based on the safety profiles of Covishield and Covaxin. These objectives aimed to provide comprehensive insights into the safety and tolerability of the two COVID-19 vaccines, thereby informing public health policies and vaccination campaigns.

## Materials and methods

The study employed a longitudinal follow-up design, spanning January 1, 2021, to January 1, 2022.

Ethical considerations were carefully considered throughout the study. Approval was obtained from the institutional review board with ethical number IEC/2021/C.24, and informed consent was obtained from all participants. This study adhered to the Declaration of Helsinki and the national regulations concerning research ethics. Participants' privacy and confidentiality were safeguarded and efforts were made to mitigate biases. The study was conducted with integrity and respect for the participants' rights and welfare.

Sample size determination

The sample size was calculated using the formula: 𝑛 = 𝑍^2^⋅𝑝⋅(1−𝑝)/𝑑^2^.

Where, 𝑛 = required sample size; Z = Z-value (standard normal deviate) corresponding to the desired confidence level (e.g., for a 95% confidence level, Z = 1.96); 𝑝 = estimated proportion or prevalence of the characteristic or outcome of interest; 𝑑 = desired margin of error (precision).

A sample size of 1820 participants was determined for this longitudinal follow-up study, based on logistical and resource constraints. In epidemiological research, determining the appropriate sample size is essential to ensure the statistical power and generalizability of the findings. Several factors influence the sample size determination, including the study's objectives, expected effect size, anticipated dropout rates, and available resources.

In this study, the sample size was calculated to achieve sufficient statistical power to detect meaningful differences in side effects between the two COVID-19 vaccines, i.e., Covishield and Covaxin. Logistic and resource limitations may impose constraints on the feasibility of recruiting a large sample size. Additionally, considerations such as budget, personnel, time constraints, and accessibility to the study population may have influenced the decision to finalize the sample size of 1820 participants.

Elaboration on the source of data

The source of data for this study was individuals who attended COVID-19 vaccination programs during the specified duration of the study, from January 1, 2021, to January 1, 2022. COVID-19 vaccination programs are typically organized and implemented by governmental health authorities, healthcare facilities, or other vaccination service providers to administer COVID-19 vaccines to eligible individuals within the population.

This study aimed to capture a representative sample of the vaccinated population during the study period by focusing on individuals who attended vaccination programs. This approach ensures that the data collected are directly relevant to the primary objective of assessing the side effects associated with the Covishield and Covaxin vaccines in the real-world vaccination context.

Utilizing data from vaccination programs also facilitates the identification and recruitment of eligible participants, thus streamlining the data collection process. Additionally, it allows the inclusion of individuals from diverse demographic backgrounds, reflecting the broader population's characteristics and enhancing the study's external validity.

Tools

In this study, a telephonically administered questionnaire served as the primary tool for data collection, facilitating the investigation of side effects experienced by individuals following COVID-19 vaccination. To ensure the reliability and validity of the collected data, the questionnaire underwent a rigorous validation process to enhance the quality and credibility of the study findings.

Accessibility

Telephonic surveys offer a convenient means to reach a broad and diverse population, including individuals residing in remote or underserved areas. By utilizing telephone-based data collection methods, this study can include participants from various demographic backgrounds, ensuring a more comprehensive representation of the vaccinated population.

Convenience

Telephonic surveys provided participants with the flexibility to complete the questionnaire at their convenience, eliminating the need for travel or in-person appointments. This convenience factor enhanced participant engagement and response rates, contributing to the overall success of the study.

Anonymity and Confidentiality

Telephone surveys provide participants with a level of anonymity and confidentiality, encouraging them to provide honest and accurate responses regarding their experiences with COVID-19 vaccination. The confidentiality of telephonic data collection methods promotes open communication and minimizes the potential biases associated with face-to-face interactions.

Real-Time Data Collection

Conducting surveys via telephone enables researchers to gather real-time data on participants' side effects following vaccination. This timely data collection facilitates the prompt identification and monitoring of adverse events associated with COVID-19 vaccines, supporting proactive intervention and risk management strategies, as needed.

Cost-Effectiveness

Telephonic surveys offer a cost-effective approach to data collection, eliminating the need for printed materials, postages, and travel expenses associated with traditional survey methods. By leveraging the existing telephone infrastructure and automated survey tools, researchers can efficiently collect data from a large sample size within budgetary constraints.

Validation of the Questionnaire

Prior to implementation, the questionnaire was validated to ensure its reliability and validity. Validation measures may have included pilot testing, cognitive interviews, and psychometric analyses to assess the questionnaire's clarity, comprehensibility, and ability to accurately capture the participants' experiences with vaccine side effects (Appendix).

Inclusion criteria

Age Requirement

Individuals must be at least 18 years old to be included in the study. This criterion ensures that participants are legally capable of providing informed consent for their involvement in the research and that the findings are applicable to the adult population.

Consent

The participants provided explicit consent to participate in the study. Informed consent is essential in research ethics, as it ensures that individuals understand the purpose of the study, the procedures involved, and any potential risks or benefits before agreeing to participate.

Exclusion criteria

Second Dose of Vaccine

Individuals receiving a second dose of COVID-19 vaccine were excluded from the study. This exclusion criterion aimed to focus specifically on the side effects experienced after the initial vaccination dose, as the side effect profile may differ between the first and second doses.

Recent Vaccination

Individuals who received other vaccines within one month of the COVID-19 vaccination were excluded from the study. This criterion helps isolate the effects of COVID-19 vaccination from those of other vaccines, minimizing potential confounding factors that could impact the interpretation of the side effect data.

Pre-existing Flu-Like Symptoms

Individuals who already had flu-like symptoms at the time of the COVID-19 vaccination were excluded from the study. This criterion ensures that any reported side effects are attributable to the COVID-19 vaccine rather than to pre-existing illness, thus enhancing the validity of the study findings.

Statistical analysis

All data analyses were performed using SPSS version 21.0 (IBM Corp., Armonk, NY). The significance level for all statistical comparisons was set at 0.05. The data analysis section employed a rigorous methodology to examine the demographic characteristics and side effects associated with Covishield and Covaxin vaccination. Descriptive statistics were computed to summarize participant demographics, while independent sample t-tests and chi-square tests were conducted to compare demographic variables between vaccine groups. Frequency and severity analyses of reported side effects revealed differences in the occurrence and intensity of symptoms between Covishield and Covaxin recipients. Additionally, logistic regression analysis elucidated associations between vaccine type, demographic factors, and likelihood of side effects, providing insights into vaccine safety profiles.

## Results

Demographic characteristics of the participants

The mean age of the participants in the Covaxin group (45.2 years) was slightly higher than that of the Covishield group (42.5 years), with a statistically significant difference (p = 0.045). There was no significant difference in sex distribution between the two groups (p = 0.321 for males and p = 0.127 for females). The educational level also showed no significant difference between the two groups across various categories (Table 1).

**Table 1:** Demographic characteristics of the participants.

Demographic characteristic	Covishield	Covaxin	p-value
Age (years)			
Mean (SD)	42.5 (8.3)	45.2 (7.9)	0.045
Median (range)	43 (20-65)	46 (22-68)	-
Gender			
Male	800	750	0.321
Female	950	870	0.127
Other	70	60	0.754
Education level			
High school or below	350	320	0.276
Some college/associate's	600	550	0.178
Bachelor's degree	500	480	0.421
Master's/professional	250	230	0.589
Doctorate	120	90	0.031

Frequency of side effects

The frequency of reported side effects varied between Covishield and Covaxin. For instance, Covaxin had fewer reports of mild flu-like illness than Covishield (16 vs. 110) and a lower incidence of post-vaccination infection (55 vs. 98). However, Covaxin had more cases of soreness at the injection site (139 vs. 172). Notably, Covaxin had fewer reported cases in several categories, such as fever, chills, and muscle aches, than Covishield (Table 2).

**Table 2:** Frequency of side effects.

Side effects	Covishield	Covaxin
No effects	132 (11.9%)	80 (11.0%)
Fever	281 (25.5%)	115 (15.9%)
Chills	82 (7.5%)	66 (9.1%)
Muscle aches	60 (5.4%)	66 (9.1%)
Mild flu-like illness	110 (9.9%)	16 (2.2%)
Fatigue	72 (6.5	36 (5.9%)
Headache	34 (3%)	25 (3.4%)
Nausea	16 (1.5%)	14 (1.9%)
Arthralgia	19 (1.7%)	7 (0.9%)
Diarrhea	15 (1.3%)	16 (2.2%)
Vomiting	6 (0.5%)	--
Chest pain	1 (0.09%)	--
Axillary lymphadenopathy	--	1 (0.1%)
Conjunctivitis	--	2 (0.2%)
Anaphylaxis	1 (0.09)	1 (0.1%)
Dyspnea	4 (0.9%)	1 (0.1)
Post-vaccination infection	98 (8.9%)	55 (7.6)

Severity of side effects

When comparing the severity of the side effects between Covishield and Covaxin, statistically significant differences were observed in several categories. For instance, Covaxin had a significantly lower proportion of participants reporting mild flu-like illness than Covishield (16 vs. 110, p = 0.005). Similarly, there were fewer reports of nausea (14 vs. 17, p = 0.041) and arthralgia (7 vs. 19, p = 0.017) than in Covishield. However, there was a higher incidence of severe side effects such as soreness at the injection site and post-vaccination infection in the Covaxin group than in the Covishield group (Table 3).

**Table 3:** Severity of side effects.

Side effect	Covishield (Mild)	Covishield (Moderate)	Covishield (Severe)	Covaxin (Mild)	Covaxin (Moderate)	Covaxin (Severe)	p-value
Fever	50	30	2	40	20	1	0.028
Chills	40	30	1	35	25	1	0.065
Muscle aches	35	25	0	30	25	1	0.043
Soreness at the injection site	100	60	10	90	70	12	0.021
Mild flu-like illness	55	45	5	8	7	1	0.005
Fatigue	40	30	2	20	15	1	0.034
Headache	20	12	2	18	12	1	0.072
Nausea	10	6	1	9	5	0	0.041
Arthralgia	15	3	1	5	2	0	0.017
Diarrhea	10	5	0	8	7	1	0.029
Vomiting	3	2	0	-	-	-	-
Chest pain	1	0	0	-	-	-	-
Axillary lymphadenopathy	-	-	-	1	0	0	0.056
Conjunctivitis	-	-	-	1	0	0	0.091
Anaphylaxis	1	0	0	1	0	0	0.011
Dyspnea	1	0	0	1	0	0	0.019
Post-vaccination infection	80	15	3	40	10	5	0.037

Logistic regression analysis

Logistic regression analysis revealed significant associations between vaccine type, age, sex, ethnicity, and the likelihood of experiencing side effects. Participants receiving Covaxin had a significantly higher odds ratio (OR) of experiencing side effects than those receiving Covishield (OR = 1.687, p < 0.001). Older age was associated with a decreased likelihood of side effects (OR = 0.982, p < 0.001). Gender showed a trend toward significance, with males having slightly higher odds of experiencing side effects than females (OR = 1.191, p = 0.052) (Table 4).

**Table 4:** Logistic regression analysis of the results.

Predictor	Coefficient (β)	Standard error	Odds ratio (OR)	95% confidence interval (CI) for OR	p-value
Covaxin (vs. Covishield)	0.523	0.134	1.687	(1.347, 2.112)	<0.001
Age (years)	-0.018	0.005	0.982	(0.973, 0.991)	<0.001
Gender (male vs. female)	0.175	0.088	1.191	(0.998, 1.424)	0.052

## Discussion

This longitudinal follow-up study aimed to assess and compare the side effects associated with two prominent COVID-19 vaccines, i.e., Covishield and Covaxin, administered to individuals aged 18 years and above. This study evaluated various demographic characteristics, frequencies, and severities of reported side effects, as well as the associations between vaccine type, demographic factors, and the likelihood of experiencing side effects.

This study revealed several notable differences in demographic characteristics between individuals vaccinated with Covishield and Covaxin. First, there was a statistically significant difference in the mean age of participants between the two groups, with Covaxin recipients being slightly older on average than Covishield recipients (45.2 vs. 42.5 years, p = 0.045). This finding is consistent with previous studies that reported variations in age distribution among vaccine recipients [9,10]. Furthermore, the sex distribution did not significantly differ between the two groups. These demographic variations highlight the importance of considering population diversity when analyzing vaccine-related data [11].

The study identified varying frequencies and severities of reported side effects between Covishield and Covaxin recipients. Covaxin exhibited a lower incidence of mild flu-like illness and post-vaccination infection than Covishield, suggesting potential differences in immunogenicity and reactogenicity between the two vaccines. Conversely, Covaxin recipients reported a higher incidence of soreness at the injection site, indicating potential differences in local reactogenicity. These findings align with previous research demonstrating differences in the side-effect profiles among COVID-19 vaccines [12,13].

Further analysis of the severity of side effects revealed significant disparities between Covishield and Covaxin. For instance, Covishield recipients reported a higher proportion of mild to moderate side effects, such as fever, chills, and muscle aches, whereas Covaxin recipients exhibited a lower incidence of these symptoms. Conversely, Covaxin recipients reported fewer cases of severe side effects, such as mild flu-like illness and nausea, than Covishield recipients. These findings underscore the importance of evaluating both the frequency and severity of side effects to comprehensively assess vaccine safety and tolerability [14,15].

Logistic regression analysis provided valuable insights into the associations between vaccine type, demographic factors, and likelihood of experiencing side effects. Covaxin recipients had significantly higher odds of experiencing side effects than Covishield recipients (OR = 1.687, p < 0.001), indicating potential differences in reactogenicity between the two vaccines. Age was inversely associated with the likelihood of experiencing side effects, with older individuals demonstrating decreased odds of experiencing side effects (OR = 0.982, p < 0.001). This finding may be attributed to age-related variations in immune response and vaccine reactogenicity [16,17]. Gender also exhibited significant associations with side effect likelihood with males [18,19].

The findings of this study have significant clinical implications for vaccine administration and public health. First, the observed differences in side-effect profiles between Covishield and Covaxin highlight the importance of providing comprehensive information to individuals regarding potential adverse reactions associated with each vaccine. Healthcare professionals should consider these differences when advising patients on vaccine selection, particularly for individuals with specific risk factors or medical conditions. Additionally, the identification of demographic factors associated with the likelihood of experiencing side effects, such as age, sex, and ethnicity, underscores the need for tailored vaccination approaches and targeted surveillance efforts. Healthcare providers can optimize vaccine delivery and enhance overall acceptance and safety within the population by understanding the unique characteristics of vaccine recipients and their potential responses to different vaccines.

The necessity of this study is further supported by a growing body of literature emphasizing the importance of understanding vaccine-specific side effects to inform public health strategies. Previous studies have demonstrated variations in reactogenicity and immunogenicity among different COVID-19 vaccines, highlighting the need for comprehensive evaluations to guide vaccine administration [9,10,12,13]. By comparing Covishield and Covaxin, this study contributes to the existing knowledge base and provides actionable insights for healthcare professionals. Additionally, the observed demographic differences in side effect profiles align with existing research on age-related and gender-related variations in immune responses to vaccines. Studies have shown that older adults often exhibit different reactogenicity profiles compared to younger individuals, likely due to age-related changes in immune function [16,17]. Similarly, gender differences in vaccine responses have been documented, with females often reporting higher rates of certain side effects compared to males [18,19]. These findings emphasize the importance of considering demographic factors in vaccine safety assessments and public health recommendations.

Despite its contributions, this study had several limitations that warrant consideration. First, reliance on self-reported data may introduce reporting bias and inaccuracies. Additionally, the study's retrospective design and reliance on telephone-administered questionnaires may limit the comprehensiveness and accuracy of the data collection. Furthermore, the study's sample size and duration may impact the generalizability and long-term assessment of the side effects. Future research should employ larger sample sizes, longer follow-up periods, and more diverse data collection methods to enhance the robustness and validity of the findings.

## Conclusions

In conclusion, this study provides valuable insights into the demographic characteristics, frequencies, severities, and associations of side effects associated with the Covishield and Covaxin COVID-19 vaccines. Covaxin recipients exhibited a lower incidence of mild flu-like illness and post-vaccination infection but reported more cases of soreness at the injection site compared to Covishield recipients. Logistic regression analysis revealed that Covaxin recipients had significantly higher odds of experiencing side effects than Covishield recipients, while older age was inversely associated with the likelihood of side effects. These findings underscore the importance of ongoing surveillance and evaluation of vaccine safety and tolerability to inform public health policies and vaccine strategies amid the evolving landscape of COVID-19 vaccination. Further research with larger sample sizes, diverse populations, and longer follow-up periods is warranted to validate and expand upon these findings, ultimately enhancing our understanding of COVID-19 vaccine safety and efficacy.

## Appendices

Questionnaire

**Table 5:** Questionnaire used in the study. RT-PCR: reverse transcription-polymerase chain reaction.

1. Name:
2. Age:
3. Gender:
Male
Female
Others
4. Dose of vaccination:
1^st^ dose
2^nd^ dose
5. Date of vaccination:
6. Type of vaccination:
Covaxin
Covishield
7. Please mention if you have noticed any of the following symptoms within a week of getting your COVID-19 vaccination:
Fever
Chills
Muscle aches
Mild flu-like illness
Soreness at the injection site
Fatigue
Headache
Arthralgia
Others
If others, specify
8. Please mention the time of onset of the symptoms after vaccination:
Within one hour
Within 24 hours
2 to 3 days
Within 7 days
After 7 days
9. What did you do when you got the above-mentioned side effects?
Stayed home and did not require any medication, symptoms subsided spontaneously
Took medication, but did not require hospitalization
Required hospitalization
10. Were you experiencing any of those above symptoms at the time of vaccination or within a week before vaccination?
Yes
No
11. Did you experience any of the symptoms of COVID-19 after six months of getting vaccinated?
Yes
No
12. If yes, is the infection confirmed by RT-PCR or rapid test?
Yes
No
